# A Feasibility Study on the Simultaneous Sensing of Turbidity and Chlorophyll *a* Concentration Using a Simple Optical Measurement Method

**DOI:** 10.3390/mi8040112

**Published:** 2017-04-01

**Authors:** Ryota Isoyama, Manami Taie, Tomoaki Kageyama, Masashi Miura, Akihiro Maeda, Akihiro Mori, Sang-Seok Lee

**Affiliations:** 1 Graduate School of Engineering, Tottori University, Tottori 680-8552, Japan; b13t3003@eecs.tottori-u.ac.jp (R.I.); b12t3032@faraday.ele.tottori-u.ac.jp (M.T.); m14t3010@faraday.ele.tottori-u.ac.jp (T.K.); contact@m-miura.jp (M.M.); 2 Environment Sanitation Research Center, Tottori 682-0704, Japan; maedaa@pref.tottori.lg.jp (A.Ma.); moriak@pref.tottori.lg.jp (A.Mo.)

**Keywords:** water quality monitoring, turbidity, chlorophyll *a* concentration, optical setup, wireless sensor network

## Abstract

We have been developing a wireless sensor network system to monitor the quality of lake water in real time. It consists of a sensor module and a system module, which includes communication and power modules. We have focused on pH, turbidity and chlorophyll *a* concentration as the criteria for qualifying lake water quality. These parameters will be detected by a microfluidic device based sensor module embedded in the wireless sensor network system. In order to detect the turbidity and the chlorophyll *a* concentration simultaneously, we propose a simple optical measurement method using LED and photodiode in this paper. Before integrating a turbidity and chlorophyll *a* concentration sensor into the microfluidic device based pH sensor, we performed feasibility studies such as confirmation of the working principle and experiments using environmental water samples. As a result, we successfully verified our simultaneous sensing method by using a simple optical setup of the turbidity and the chlorophyll *a* concentration.

## 1. Introduction

In terms of natural resource preservation, a real-time and constant environmental monitoring system is an important research and development topic [1,2,3,4,5,6]. The targets of the environmental monitoring systems are very far-reaching and can include air, water, and soil. In this paper, we specifically focus on water quality monitoring, which is one of the most important issues worldwide. Water quality is closely related to the quality of drinking water and water for fishing and agriculture. In typical water quality monitoring systems, physical and chemical quantities such as pH, turbidity, dissolved oxygen concentration, chlorophyll *a* concentration, temperature, salinity, and pressure are monitored. However, a commercially available conventional water quality monitoring system is generally large and expensive. The high cost of monitoring systems prevents the real-time and continuous monitoring of a large area. In other words, sufficient numbers of water quality monitoring systems cannot be established in large natural resources such as lakes or rivers due to their high cost. It also causes low accuracy of water quality monitoring.

In order to obtain a low cost and high accuracy for lake or river water quality monitoring systems, we have been developing a wireless sensor network system. Our wireless sensor network system consists of a sensor module and a system module, which includes a wireless transceiver module, control ICs for sensors, and a power module. Previous partial development results on the nodes of wireless sensor network systems have been reported in [7]. On the other hand, in the case of the sensor module, various sensors to monitor water quality, such as pH sensors, turbidity sensors, chlorophyll *a* concentration sensors, and temperature sensors, will be integrated into a microfluidic device. Among those sensors, we have developed a pH sensor. Our microfluidic device-based pH sensor measures pH with three electrodes: working, counter, and reference electrodes. We integrated three electrodes into the microfluidic channel, including a reference electrode. This can be applicable to not only water quality monitoring but also other fields such as nanoparticle synthesis monitoring [8]. Before integrating the turbidity and chlorophyll *a* concentration sensors into the microfluidic sensor module with a pH sensor, we studied the feasibility of simultaneous sensing through a simple optical measurement setup. Our optical measurement setup, using LEDs and photodiodes (PDs), is simple and costs little, whereas the commercial turbidity and chlorophyll *a* concentration sensors use expensive optical systems. Moreover, our measurement setup allows us to combine two sensors into one. Although many commercial turbidity and chlorophyll *a* concentration sensors require large samples or should directly contact samples due to their probe size or structure, respectively, our measurement setup does not require sample quantities that are as large, and we can detect turbidity and chlorophyll *a* concentration without contact. Our setup also provides more exact results in the calibration.

In this paper, we propose a simple optical measurement method for the simultaneous sensing of turbidity and chlorophyll *a* concentration, and report the results of a feasibility study.

## 2. Optical Measurement Setup

We propose a simple optical measurement method for the simultaneous sensing of turbidity and chlorophyll *a* concentration of the lake water. Basically, we obtain these two quantities with a single measurement setup by measuring the output light intensity, which is transmitted through the sample water. However, the physical origin of the output light to be detected is different when turbidity and chlorophyll *a* concentration measurements are taken. In the case of turbidity sensing, we detect the scattered light intensity caused from the suspended tiny pollutant particles in the water. In other words, the stronger the scattered light intensity is, the higher the degree of pollution will be. On the other hand, in the case of chlorophyll *a* concentration sensing, we detect the fluorescent light intensity emitted from chlorophyll *a* molecules. Chlorophyll *a* molecules emit fluorescence with a wavelength of around 685 nm when an excitation with a wavelength of around 450 nm is applied [9,10]. If the suspended aquatic phytoplankton is rich, then chlorophyll *a* concentration is high and the fluorescence intensity becomes strong.

**Figure 1:**
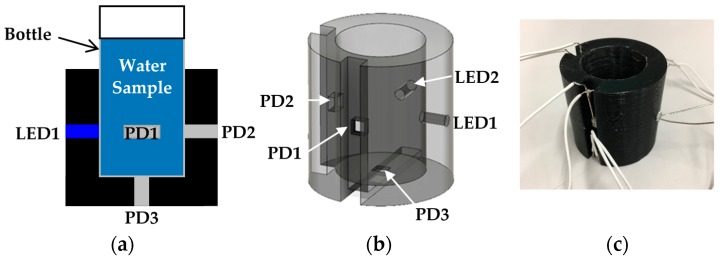
Optical measurement setup for turbidity and chlorophyll *a* concentration sensing: (**a**) schematic diagram of the optical measurement setup; (**b**) detailed structure of the socket; (**c**) photograph of a 3D printed socket with wires for measurement.

A schematic diagram of the optical measurement setup is shown in Figure 1. In the optical measurement setup, we utilized low cost LEDs and photodiode (PD) as an incident light source and a detector of the transmitted light intensity, respectively. The small water sample bottle (diameter; 24 mm, height including lid; 52 mm) is put into the socket and fixed. The socket was fabricated by a 3D printing technique. As shown in Figure 1, in the socket, we prepared 5 holes for 2 LEDs and 3 PDs to investigate an optimal measurement position. However, generally, we use 2 LEDs such as a red and a blue LED. These 2 LEDs and 1 PD are sufficient to measure two quantities in real time.

In the experiment, we used the red LED with a typical wavelength of 624 nm (OS5RKA3131A, OptoSupply, Hong Kong, China) and the blue LED with a typical wavelength of 470 nm (OSB56A3131A, OptoSupply) as incident light for turbidity sensing and an excitation light for chlorophyll *a* fluorescence, respectively. The PD (S7183, Hamamatsu Photonics, Hamamatsu, Japan) with a spectral response range of 300–1000 nm was adopted. The diameter of the LEDs, and the area including the burr of the PD are 3 mm and 4.3 × 4.6 mm^2^, respectively.

**Figure 2:**
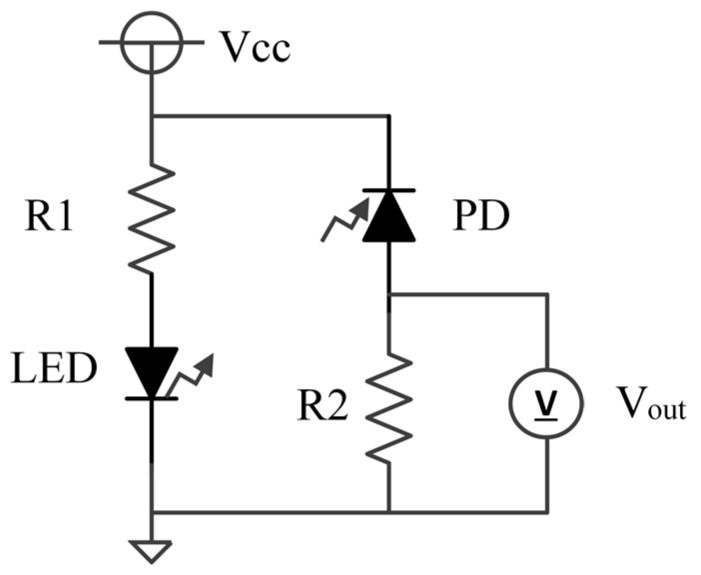
Equivalent circuit diagram of the optical measurement setup: The LED represents an LED1 or an LED2 in Figure 1b.

**Figure 3:**
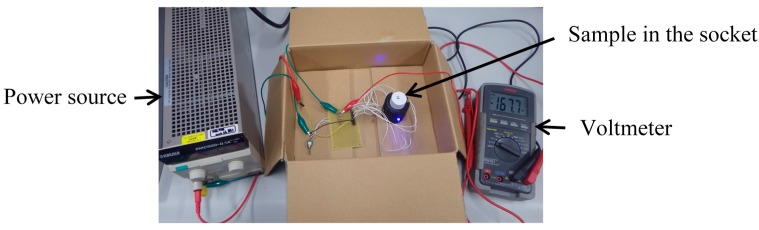
A view of the experiment with the optical measurement setup based on the circuit configuration shown in Figure 2.

An equivalent circuit diagram of our optical measurement setup is shown in Figure 2. The power supply voltage (Vcc), the current control resistor (R1) for the LEDs, and the bias resistor (R2) for photovoltage measurement are 5 V, 0.984 kΩ, and 9.89 kΩ, respectively. In the experiment, a socket with the sample is placed in a box to prevent the influence of environmental light. A view of the experiment is shown in Figure 3. The measurement system is assembled based on the equivalent circuit shown in Figure 2. Moreover, in the experiment, we performed turbidity and chlorophyll *a* concentration sensing experiments individually to demonstrate feasibility.

## 3. Experiment Results and Discussions

We performed the turbidity and chlorophyll *a* concentration sensing experiments with standard samples for calibration and the environmental water samples to demonstrate the feasibility as follows, before performing the integration of two sensors into a microfluidic pH sensor.

### 3.1. Turbidity Sensing

Before the turbidity sensing experiment, we performed calibration to clarify the correlation between turbidities and output photovoltages. In accordance with international standard ISO 7027 [11], a formazine solution is recommended as the turbidity standard solution for measuring turbidity by the optical method. However, formazine is difficult to handle because a hydrazine sulfate, which is a carcinogenic agent, is necessary in the mixing process to obtain the formazine solution [12]. On the other hand, kaolin has been prevalently utilized as a turbidity standard solution for a long time. Since kaolin is a clay mineral, it is completely free of anything harmful, is easy to handle, and is a low-cost material. However, a kaolin solution settles easily. It causes variations in turbidity during the measurement. In order to resolve the problem, a styrene-divinylbenzene (St-DVB) copolymer microbeads solution was proposed as the turbidity standard solution. However, a St-DVB copolymer microbeads turbidity standard solution is much more expensive than other turbidity standard solutions. In the calibration, we utilized both a kaolin solution and a St-DVB copolymer microbeads solution.

**Figure 4:**
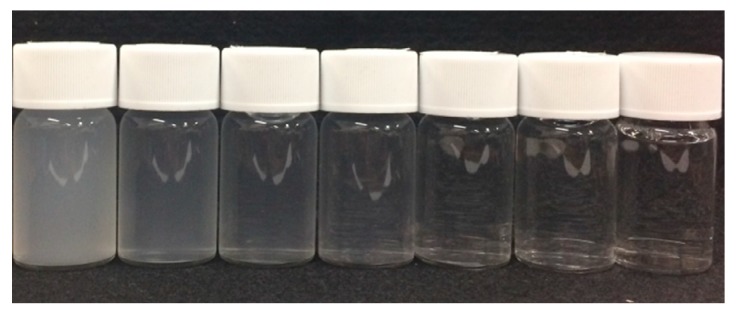
The 7 different concentration calibration solutions of kaolin: From left to right, the concentrations are 500, 200, 100, 50, 20, 10, and 0 mg/L.

**Figure 5:**
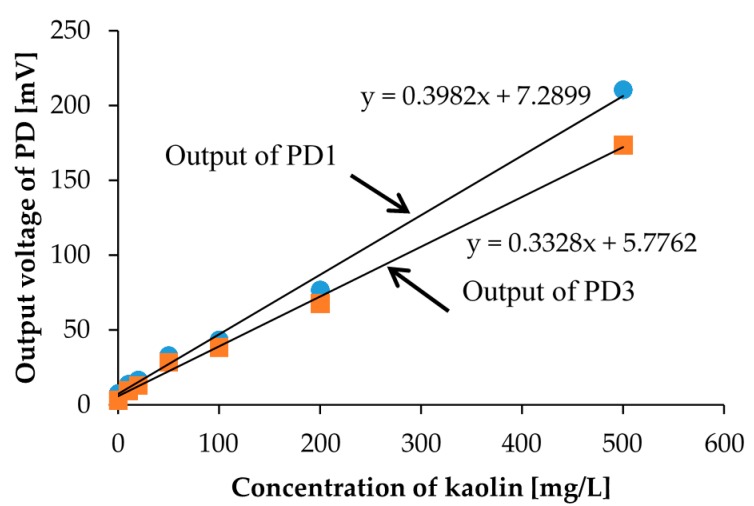
The measurement results for the calibration solutions of kaolin shown in Figure 4. PD1 is placed perpendicular to LED1, and PD3 is placed at the bottom of the sample.

First, we prepared 7 different concentration calibration solutions of kaolin. The prepared kaolin concentrations are 0, 10, 20, 50, 100, 200, and 500 mg/L, as shown in Figure 4. Then, we measured the photovoltages for each kaolin solution with the optical measurement setup shown in Figure 3. In the measurement, the red LED was set to the LED1 position in Figure 1b, and we measured the output photovoltages at three positions: PD1, PD2, and PD3, also shown in Figure 1b. The measurement results are represented in Figure 5. The photovoltages measured at the position of PD2 are not represented in Figure 5 because the position of PD2 is aligned with LED1 and because the photovoltages were out of range. In other words, PD2 measured the light intensity of the light source LED1 rather than that of the scattered light caused from the suspended kaolin particles. However, if the sample is a dense suspension or if the LED and PD are far away from each other, the measurement with LED1 and PD2 may also be valid.

As a result, we were able to distinguish the degrees of turbidity according to the measured photovoltages, but we could not distinguish them with the naked eye, especially for low concentration samples shown in Figure 4. Furthermore, we were also able to confirm the linear relationship between the concentration of the calibration solution and the scattered light intensity. The linearity was confirmed for the positions of both PD1 and PD3. We observed more accurate linearity in the measurement results from PD3. This linearity comes from the rapid precipitation effect of kaolin in the measurement, which means that the photovoltage measurement at the bottom of the sample yields a more accurate turbidity. Moreover, especially in low concentration regions of less than 50 mg/L, which is a region that is more meaningful for the sensors during detection, it shows better linearity. In turbidity sensing with environmental water samples, zero calibration is necessary only when the scattered light intensity is considered.

**Figure 6:**
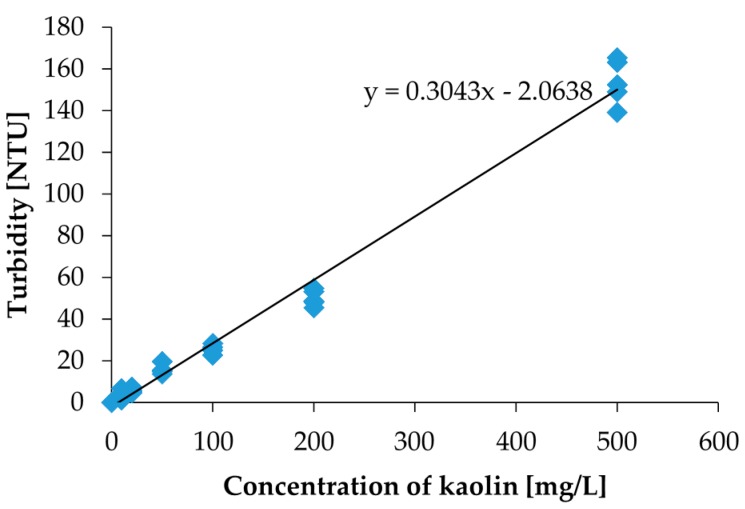
The measurement results by the commercial turbidity sensor for the same kaolin calibration solutions used in the measurement by the measurement setup in Figure 3. The photovoltage values can be converted to an NTU value.

To convert the photovoltage value to the Nephelometric Turbidity Unit (NTU), we measured the same kaolin calibration solutions with a commercial turbidity sensor (Hydrolab DS5X, OTT Hydromet GmbH, Kempten, Germany) calibrated with a formazine calibration solution. The NTU is defined by the formazine calibration solution. The turbidity measurement results are shown in Figure 6. Using the measurement results in Figure 6, the measured photovoltages were converted to NTU turbidities.

**Figure 7:**
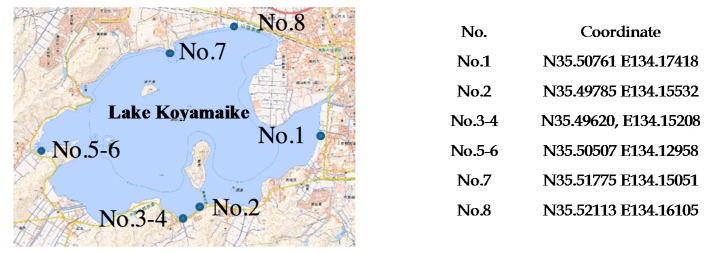
Sampling position at Lake Koyamaike in Tottori Prefecture, Japan. We collected 8 environmental water samples from Lake Koyamaike.

**Figure 8:**
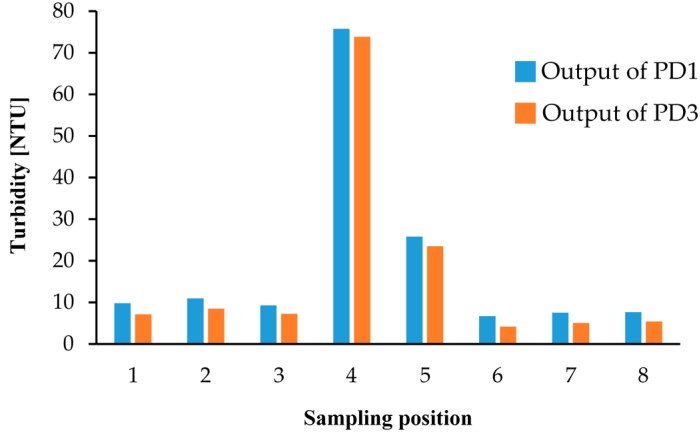
Turbidity measurement results for samples at the 8 places indicated in Figure 7.

We also performed turbidity measurements with environmental water samples from Lake Koyamaike located in the Tottori Prefecture of Japan. We collected eight measurement samples in Lake Koyamaike, and the places are indicated in Figure 7. Sample Nos. 3 and 4, and Nos. 5 and 6 were collected at the same place, but Sample Nos. 4 and 6 were collected after a pause. In the measurement, we measured the photovoltage for each sample, and it was converted to NTU turbidity using the relationship shown in Figure 6. The NTU turbidity measurement results are summarized in Figure 8. The photovoltage measurements were performed by PD1 and PD3, and the results were in agreement. The turbidity of Sample Nos. 4 and 5 are relatively higher than the others due to the muddiness of the stream during sampling.

**Figure 9:**
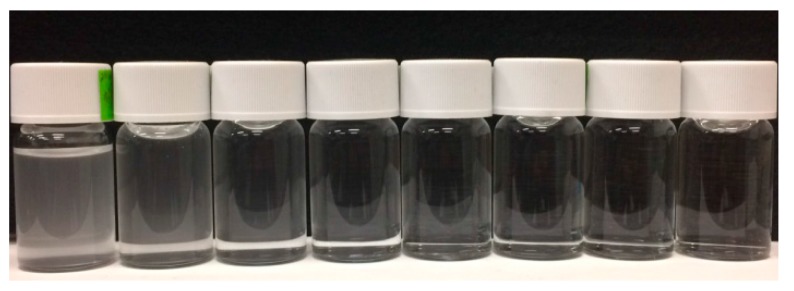
The 8 different concentration calibration solutions of St-DVB copolymer microbeads: From left to right, the concentrations are 100, 50, 20, 10, 5, 2, 1, and 0 mg/L.

**Figure 10:**
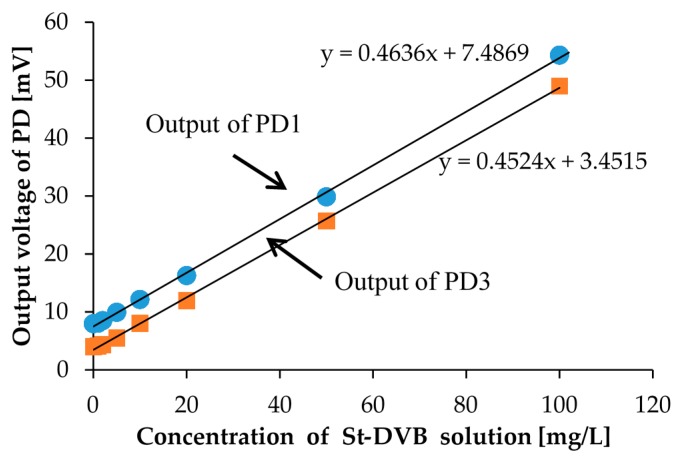
The measurement results for calibration solutions of St-DVB copolymer microbeads shown in Figure 9. PD1 is placed perpendicular to LED1, and PD3 is placed at the bottom of the sample.

We also performed calibration with the St-DVB copolymer microbeads solutions for comparison. For calibration solutions of St-DVB copolymer microbeads, we prepared eight different concentration calibration solutions. The prepared St-DVB copolymer microbeads solution concentrations were 0, 1, 2, 5, 10, 20, 50, and 100 mg/L and are shown in Figure 9. In the measurement, the measurement setup and procedure were the same as that used for the kaolin calibration solutions. The measurement results are shown in Figure 10. Here, we only measured the photovoltages from PD1 and PD3.

With the St-DVB copolymer microbeads calibration solution, we were also able to distinguish the degrees of turbidity according to the measured photovoltages. Moreover, we were able to successfully confirm the linear relationship between the concentration of the calibration solution and the scattered light intensity. Although the measurement results between PD1 and PD3 have small differences, we could obtain linearity with almost identical slopes. This means that the St-DVB copolymer microbeads are uniformly distributed in the solution and the precipitation effect can be ignored. The zero calibration is also necessary only to consider the scattered light intensity when we measure the turbidity of the environmental water sample.

**Figure 11:**
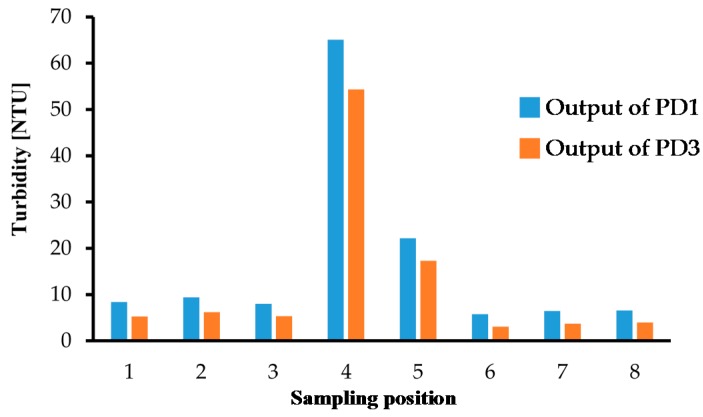
Turbidity measurement results based on St-DVB copolymer microbeads solution calibration for samples at the 8 places indicated in Figure 7.

We also performed turbidity measurements with the same environmental water samples, which were used in previous turbidity measurements. The measurement results based on the St-DVB copolymer microbeads solution calibration are shown in Figure 11. The NTU turbidity conversion was performed by using the relationship shown in Figure 6 as well. The turbidity values measured by PD1 agreed with those measured by PD3. Although the obtained turbidity values based on the St-DVB copolymer microbeads solution calibration were slightly smaller than those based on kaolin solution calibration. Specifically, in most low turbidity areas, the turbidity showed almost the same values. According to the measurement results for each calibration solution shown in Figure 5 and Figure 10, the St-DVB copolymer microbeads solution calibration is considered as the more accurate method. However, in real applications, it is more important and meaningful to detect and clarify low turbidity water instead of high turbidity water. Therefore, the cheaper and easier method using the kaolin calibration solution is considered to be the effective one.

### 3.2. Chlorophyll *a* Concentration Sensing

**Figure 12:**
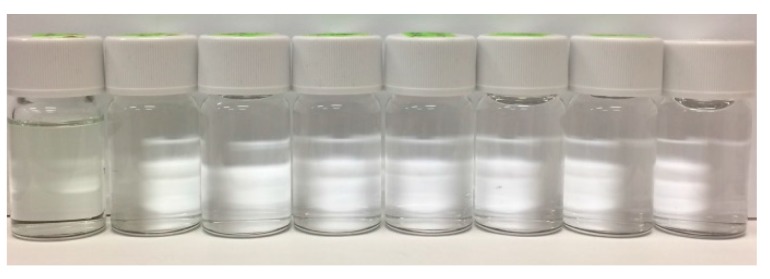
The 8 different concentration calibration solutions of chlorophyll *a*: From left to right, the concentrations are 500, 200, 100, 50, 20, 10, 5, and 0 µg/L.

**Figure 13:**
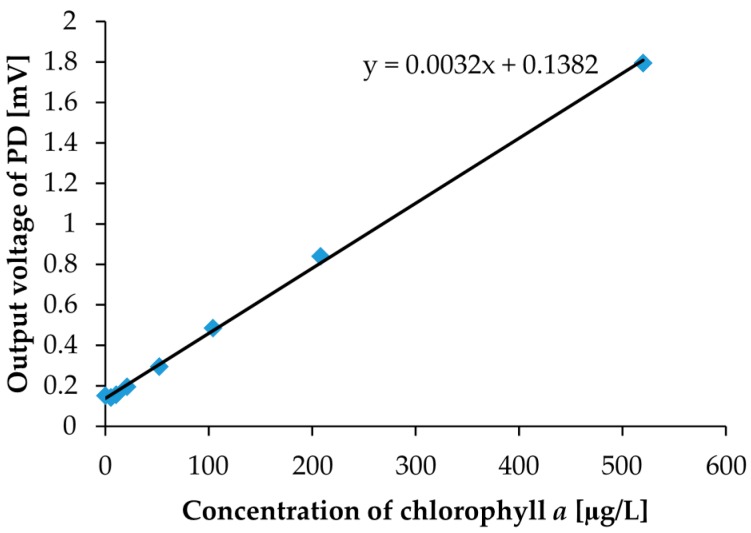
The measurement results for chlorophyll *a* solutions shown in Figure 12. PD3 placed at the bottom of the socket was used for detection.

We also performed a chlorophyll *a* concentration sensing experiment with the same optical measurement setup shown in Figure 3. Before the measurement with an environmental water sample, we calibrated to clarify the correlation between chlorophyll *a* concentrations and output photovoltages. As calibration solutions of chlorophyll *a*, we prepared eight different concentration calibration solutions. The prepared chlorophyll *a* concentrations were 0, 5, 10, 20, 50, 100, 200, and 500 µg/L, which are shown in Figure 12. With the naked eye, the concentration differences cannot be distinguished. In the measurement, the measurement setup and procedure are the same as what we used for the turbidity measurement, except for the LED. For chlorophyll *a* measurements, we utilized a blue LED instead of a red LED for the excitation of fluorescence. The blue LED was set to the LED2 position in Figure 1b. The measurement results are shown in Figure 13. In the measurement, the photovoltages were detected by PD3 only because detection by PD3 yielded better results in turbidity measurement. Moreover, we set a red filter sheet on PD3 to consider only the fluorescence around 685 nm in the measurement. For convenience in the red filter setup, we chose PD3 for the detection. As a result, we were also able to obtain good linearity between chlorophyll *a* concentrations and photovoltages. In other words, with the same simple optical measurement setup, we can easily detect both the turbidity and the chlorophyll *a* concentration. Commonly used commercial chlorophyll *a* sensors measure water soluble uranine (fluorescein disodium salt, C_20_H_10_Na_2_O_5_) fluorescence intensity for calibration, and measurement results are then converted to chlorophyll *a* concentration. However, by using our measurement setup, we can directly calibrate with the chlorophyll *a* that is dissolved in ethanol and diluted with water. In chlorophyll *a* concentration sensing with an environmental water sample, zero calibration is necessary to consider fluorescence intensity only.

**Figure 14:**
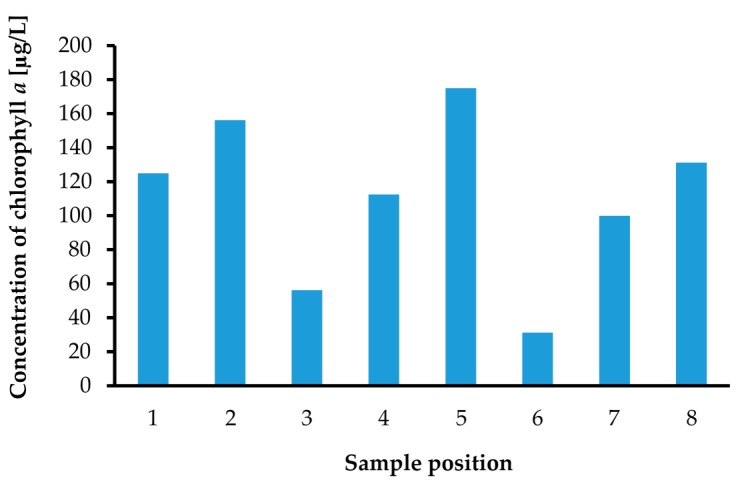
Chlorophyll *a* concentration measurement results for samples at the 8 places indicated in Figure 7.

We performed chlorophyll *a* concentration measurements with the same environmental water samples, which were used in previous turbidity measurements. The measured chlorophyll *a* concentrations are summarized in Figure 14. The chlorophyll *a* concentration was measured successfully with the same optical measurement setup applied to turbidity sensing. The chlorophyll *a* concentration is also one of the more important quantities in defining water quality. For instance, the turbidity of Sample No. 4 showed the highest value, but the chlorophyll *a* concentration of it was not high. This means that the environmental water sample at the Sample No. 4 position was only muddy and not polluted by phytoplankton.

**Figure 15:**
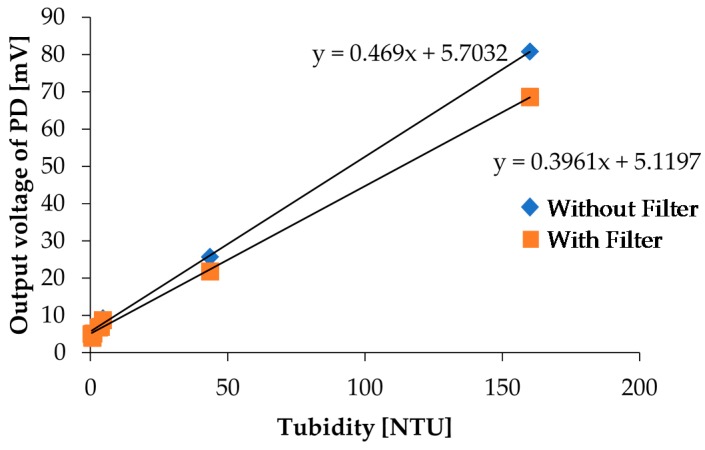
Comparison of the turbidity measurement results with and without a red filter on PD3.

In the chlorophyll *a* concentration measurement, we used a red filter in front of PD3 to effectively detect fluorescence. PD3 is also used for turbidity detection, and we performed the turbidity measurement again with a red filter on PD3 to investigate influence of the red filter installation on turbidity measurements. In the measurements, we measured photovoltages and NTU turbidities for kaolin calibration solutions shown in Figure 4 by using our measurement setup and a commercial sensor, respectively. The measurement results with and without the red filter on PD3 are shown in Figure 15. Although the output photovoltages are decreased due to scattered light intensity reduction for the high concentration sample, the linearity still exists; specifically, there are no remarkable variations for low concentration samples, which are samples that are more useful in real applications. As a result, it reveals that two LEDs and one PD are sufficient to simultaneously detect both the turbidity and chlorophyll *a* concentration with our measurement setup.

**Figure 16:**
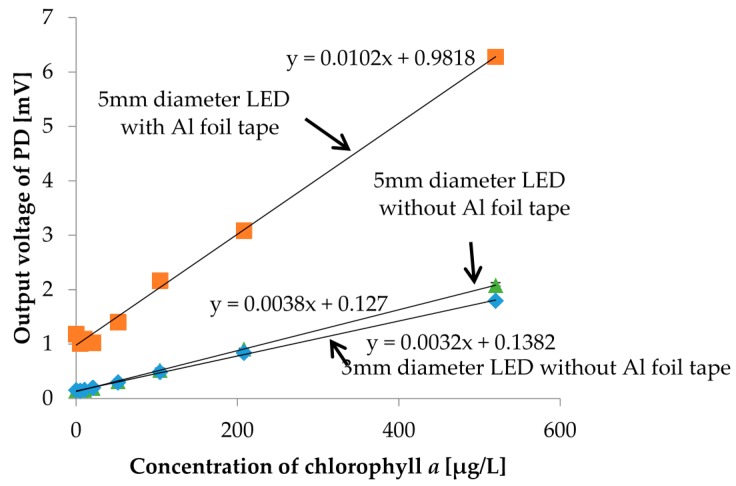
Comparison of the turbidity measurement results with and without a red filter on PD3.

**Figure 17:**
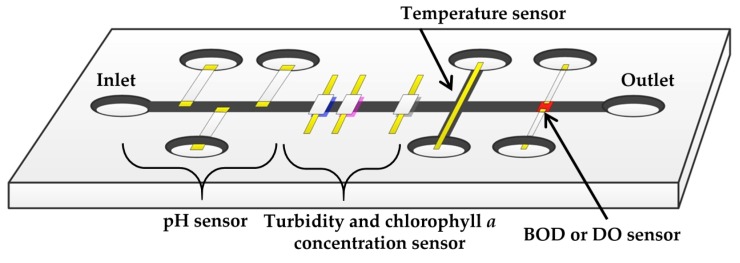
A schematic diagram of a microfluidic sensor module for the lake water quality monitoring wireless sensor network system.

In order to improve the strength of output photovoltages, we investigated the reflection effect of the socket. We put Al foil tape inside and around the socket to gather reflected scattered or fluorescent light to increase the output photovoltage. In the measurement, we used chlorophyll *a* solutions as samples shown in Figure 12. Moreover, a larger LED which has a 5 mm diameter was also tested. The measurement results are shown in Figure 16. Of course, all measurement results showed good linearity. There were no remarkable changes in the strength of photovoltages between 3 mm and 5 mm diameter LEDs. However, the output photovoltage strength was much more improved when the reflective Al foil tape was applied. For instance, for the chlorophyll *a* concentration of the 5 µg/L sample, which is sample with the lowest concentration, the output photovoltage strength was increased by 14.9%. It is obvious that using reflection in the measurement setup is effective and should be considered when turbidity and chlorophyll *a* concentration sensors are integrated into a microfluidic sensor module, as shown in Figure 17.

In environmental water measurement, it is important to guarantee constant LED intensity. To do so, the ageing effect of LED intensity must be clarified, which will be considered as a follow-up to this work. Moreover, the intensity of the LEDs and the sensitivity of the PD should be optimized to obtain an optimal device size.

## 4. Conclusions

We have been developing a wireless sensor network system to monitor lake water quality in real time. It consists of a microfluidic sensor module and a system module. In this paper, we propose a simple optical measurement setup to detect turbidity and chlorophyll *a* concentration at the same time. Before integrating it into a microfluidic sensor module, we performed feasibility studies to confirm the working principle and to obtain guidance for an optimal device design.

In the feasibility study with the optical measurement setup, we investigated the turbidity and chlorophyll *a* sensing principles. We prepared calibration solutions for each sensing experiment, and performed measurements with them. As a result, we could confirm good linearity for both turbidity and chlorophyll *a* concentration sensing. Moreover, we performed measurements with environmental water samples and we could successfully distinguish the turbidity and chlorophyll *a* concentration for each sample. Finally, the red filter effect on PD and the reflection effect of the socket were also investigated and clarified.

In the near future, turbidity and the chlorophyll *a* concentration sensors using an optical measurement setup will be integrated into microfluidics-based pH sensors.
